# Exploring eight-year trajectories of diet-related environmental pressures in the NutriNet-Santé cohort

**DOI:** 10.1038/s41598-025-29786-6

**Published:** 2025-12-06

**Authors:** Elie Perraud, Aurélien Chayre, Sylvaine Berger, Annabelle Richard, Hafsa Toujgani, Justine Berlivet, Mathilde Touvier, Benjamin Allès, Serge Hercberg, Denis Lairon, Philippe Pointereau, Hélène Fouillet, François Mariotti, Julia Baudry, Christian Couturier, Emmanuelle Kesse-Guyot, TRANSFood Consortium, Helene Charreire, Thierry Feuillet, Jean-François Huneau, Laurent Muller, Sabrina Teyssier, Juhui Wang

**Affiliations:** 1 Center of Research in Epidemiology and StatisticS (CRESS), Nutritional Epidemiology Research Team (EREN), Université Sorbonne Paris Nord and Université Paris Cité, INSERM, INRAE, CNAM, 93017 Bobigny, France; 2 Solagro, Voie TOEC Toulouse, Toulouse, France; 3 Aix Marseille Université, Inserm, INRAE, C2VN, 13005 Marseille, France; 4 UMR PNCA, AgroParisTech, INRAE, Université Paris-Saclay, Palaiseau Cedex, France; 5 MoISA, Univ Montpellier, CIRAD, CIHEAM-IAMM, INRAE, Institut Agro, IRD, Montpellier, France; 6 Univ Paris Est Créteil LabUrba, Créteil, 94010 France; 7 Université de Caen Normandie, UMR 6266 IDEES CNRS, Caen, France; 8 University Grenoble Alpes, CNRS, GAEL, INRAE, Grenoble INP, 38000 Grenoble, France

**Keywords:** Greenhouse gas emissions, Environment, Food consumption, Trajectories, Sustainability, Biodiversity, Environmental impact, Epidemiology, Nutrition

## Abstract

Few studies have explored individual diet-related environmental pressure changes, beyond greenhouse gas emissions (GHGe) and land occupation (LO). This study evaluates the trajectories in several environmental impacts of diets among 8,905 French adults from the NutriNet-Santé cohort, who completed food frequency questionnaires (distinguishing organic vs. conventional foods) in 2014, 2018, and 2022. Six environmental indicators—GHGe, LO, energy demand, ecological infrastructure use, water use, and pesticide use—were estimated at the farm perimeter using a multi-source approach. Latent class models identified trajectories for a composite environmental pressure index (EPI) and each indicator, and mixed models adjusted for energy intake and sex modeled the trajectories. On average, most environmental pressures decreased over time (e.g., GHGe by -12%, water use by -1%), indicating a general improvement. However, ecological infrastructure also declined (-9%), which represents a negative outcome as it is linked to biodiversity. Two to four trajectory profiles were identified per indicator, with most individuals showing stable or modestly decreasing trends. Four EPI trajectories emerged: increasing EPI profiles were associated with higher meat consumption, whereas decreasing EPI reflected shifts toward more plant-based diets. Despite potential awareness of the importance of sustainable diets, this study reveals that most individuals show moderate improvements in their diet-related environmental pressures, with only a very small fraction showing important decreases.

**Supplementary Information:**

The online version contains supplementary material available at 10.1038/s41598-025-29786-6.

## Introduction

Six of the nine planetary boundaries have already been crossed (climate change, novel entities, biosphere integrity, land system change, freshwater change and biogeochemical flows)^1^. In recent decades, environmental degradation due to anthropogenic activities accelerated, leading to climate disruption, the sixth mass extinction of biodiversity, and intensified water use. For example, the global surface temperature was already 1.5 °C above the pre-industrial period in 2024^2^.

Global food systems, from field to plate, are responsible for a significant part of these deteriorations, especially at the production level^3,4^. They are responsible for about one-third of the greenhouse gas emissions (GHGe)^5^, half of land use, 70% of freshwater withdrawals, and 78% of global ocean and freshwater pollution^6^. Food production is also responsible for the decline of biodiversity^7^. Environmental pressures associated with industrial food systems and agricultural practices also include biodiversity loss, notably due to monocultures, production of limited crop types and animal breeds, land-clearing for grazing and feed crops, and increased use of fertilizers and pesticides^7^. For example, extensive use of pesticides have been responsible for 40% of insect species threatened with extinction^8,9^.

The variability of dietary patterns has been linked to important variations in environmental pressures^10–12^. A recent study compared mean GHGe values of different European countries collected between 2010 and 2018, ranging from 4.0 (Spain) to 6.5 kgCO_2_eq/day/person (France)^13^. Similar figures were reported for land use, from 5.0 (Spain) to 8.2 m^2^year/day/person (France)^13^. In the EPIC-Oxford cohort, a vegetarian diet has been linked to 3.3 kgCO_2_eq/day, compared to 5.3 kgCO_2_eq/day for medium meat-eaters. Similarly, in the Unite-Kingdom, in comparison to diets of medium meat-eaters, diets of vegetarians need approximately 50% less land use, 30% less water use, 40% less for eutrophication, and have a 25% lower impact on biodiversity^11^. Similar results were found in an study from Ireland where pescatarian,vegetarian, vegan and vegetable-focused dietary pattern had the lowest GHGe, land use and water use^14^. This wide diversity of environmental pressures between diets has also been reported in French populations^15,16^. In addition to dietary patterns, farming practices have also been associated with environmental pressures^17,18^. For instance, organic farming led to lower energy demand than the conventional system. However, results are not consistent regarding its impact on GHGe^19,20^.

However, the recent dietary changes and their long-term impacts regarding environmental pressures, are poorly documented. Few studies have documented how changes in GHGe relate to the evolution of food group consumption^21–24^. For instance, a Swedish study evaluating change in food-related GHGe between 2001–2004 and 2014–2018 reported a decrease over time at the individual level, in particular among the youngest age group (−30%)^22^.

Nevertheless, it remains unknown whether the evolution of various diet-related environmental impacts, beyond GHGe, is homogeneous or varies among individuals, with distinct subgroups following different trajectories based on their dietary changes. Overall, few studies have investigated how environmental pressures from individual diets change over time.

This study aimed to describe the evolution patterns of various environmental pressures at three time points (2014, 2018, 2022) and to describe these patterns in terms of dietary characteristics in a large French cohort. This will enable to address the current lack of longitudinal analyses of diet-related environmental pressures and to expand the focus beyond GHGe to include other environmental impacts.

## Results

A comparison of the sample with subjects who completed the 1 st FFQ without data in 2018 and 2022 is presented in Supplemental Table 1. Compared to excluded participants, those in this study tended to be older, less educated, and more frequently non-smokers. Some differences were observed regarding environmental pressures, such as CED, WU, and pesticide use. These factors were higher among included participants compared to excluded ones.

### Characteristics of the sample

The description of the overall sample is presented in Table 1. The majority of the participants were women (70%), and the average age was 56 (SD = 12). In the entire sample, most of the environmental pressures decreased, as follows: by 11.7% for GHGe, by 8.2% for CED, by 11.5% for LO, by 0.5% for WU, and by 8.4% for pesticide use. However, a reduction of ecological infrastructures by 11.9% has also been observed. These infrastructures are landscape features that support biodiversity and ecosystem services, so their decline indicates an increase in this environmental pressure. However, when ecological infrastructures were related to the LO of the diets, these infrastructures decreased by only 1.7%. The means and standard deviation and the means adjusted for sex and energy intake are presented in Table 2. The EPI is highly correlated to CED (r = 0.96), GHGe (r = 0.91), LO (r = 0.86), pesticide use (r = 0.85), and ecological infrastructures (r = 0.78) but not with WU (r = 0.39) (Supplemental Fig. 2). Consequently, in the total sample, the aggregated score, EPI, decreased by 9.1% between 2014 and 2022.

**Table 1:** Baseline sociodemographic, lifestyle, and anthropometric characteristics across clusters identified using the EPI (NutriNet-Santé study, N = 8,905, 2014) ^1^.

		**Total**	**Trajectory profile 1 (Decreasing EPI)**	**Trajectory profile 2 (Increasing EPI)**	**Trajectory profile 3 (Stable mean EPI)**	**Trajectory profile 4 (Stable low EPI)**	**P-value**
**N (%)**		8905	151 (1.7)	41 (0.5)	8421 (94.6)	292 (3.3)	
**Age (y)**		56.2 (11.9)	54.1 (11.9)	54 (13.4)	56.3 (11.8)	54.7 (12.3)	< 0.01
**Sex, (%)**	Men	2663 (29.9)	40 (26.5)	12 (29.3)	2525 (30.0)	86 (29.5)	0.82
	Women	6242 (70.1)	111 (73.5)	29 (70.7)	5896 (70.0)	206 (70.5)	
**Energy Intake in 2014 (kcal/d)**		2004.2 (593.8)	2103.4 (570.9)	1985.4 (670.1)	2003.9 (591.6)	1963.1 (653.1)	0.13
**Energy Intake in 2022 (kcal/d)**		2014.2 (606.7)	2081.8 (757.3)	2334.81 (582.0)	2012.61 (601.6)	1979.22 (657.7)	< 0.01
**% of organic food in the diet**		30 (27)	30 (26)	29 (32)	28 (26)	67 (28)	
**Tobacco use, (%)**	Never-smokers	4377 (49.2)	82 (54.3)	20 (48.8)	4114 (48.9)	161 (55.1)	0.1
	Former smokers	3739 (42.0)	55 (36.4)	16 (39.0)	3551 (42.2)	117 (40.1)	
	Current smokers	789 (8.9)	14 (9.3)	5 (12.2)	756 (9.0)	14 (4.8)	
**Physical activity, (%)**	High	3117 (35.0)	50 (33.1)	14 (34.1)	2951 (35.0)	102 (34.9)	0.98
	Moderate	3245 (36.4)	57 (37.7)	14 (34.1)	3068 (36.4)	106 (36.3)	
	Low	1661 (18.7)	33 (21.9)	8 (19.5)	1567 (18.6)	53 (18.2)	
	Missing data	882 (9.9)	11 (7.3)	5 (12.2)	835 (9.9)	31 (10.6)	
**BMI in 2014 (kg/m**^**2**^**)**		24.2 (4.3)	23.6 (4.1)	27.7 (6.7)	24.3 (4.3)	21.9 (3.3)	< 0.01
**BMI in 2022 (kg/m**^**2**^**)**		24.3 (4.5)	23.5 (4.1)	26.9 (6.4)	24.4 (4.5)	21.9 (3.5)	< 0.01
**Education, (%)**	< High school diploma	1851 (20.8)	30 (19.9)	9 (22.0)	1764 (20.9)	48 (16.4)	0.62
	High school	1289 (14.5)	25 (16.6)	6 (14.6)	1217 (14.5)	41 (14.0)	
	Post-secondary graduate	5765 (64.7)	96 (63.6)	26 (63.4)	5440 (64.6)	203 (69.5)	
**Occupation, (%)**	Retired	3748 (42.1)	45 (29.8)	16 (39.0)	3586 (42.6)	101 (34.6)	< 0.01
	Executive or higher intellectual profession	1882 (21.1)	40 (26.5)	5 (12.2)	1764 (20.9)	73 (25.0)	
	Craftsman, trader, business manager, farmer	130 (1.5)	3 (2.0)	0 (0)	121 (1.4)	6 (2.1)	
	Intermediate occupation	1317 (14.8)	22 (14.6)	5 (12.2)	1245 (14.8)	45 (15.4)	
	Employee	1003 (11.3)	26 (17.2)	4 (9.8)	940 (11.2)	33 (11.3)	
	Unemployed	271 (3.0)	5 (3.3)	6 (14.6)	245 (2.9)	15 (5.1)	
	Others without professional activity	405 (4.5)	6 (4.0)	3 (7.3)	385 (4.6)	11 (3.8)	
	Student	73 (0.8)	2 (1.3)	1 (2.4)	66 (0.8)	4 (1.4)	
	Worker	76 (0.9)	2 (1.3)	1 (2.4)	69 (0.8)	4 (1.4)	
**Residential area, (%)**	Rural area	2024 (22.7)	25 (16.6)	10 (24.4)	1923 (22.8)	66 (22.6)	0.36
	Urban area < 20,000	1394 (15.7)	19 (12.6)	4 (9.8)	1326 (15.7)	45 (15.4)	
	Urban area 20,000—200,000	1632 (18.3)	32 (21.2)	6 (14.6)	1531 (18.2)	63 (21.6)	
	Urban area > 200,000	3855 (43.3)	75 (49.7)	21 (51.2)	3641 (43.2)	118 (40.4)	
**Monthly income per household unit, (%)**	< 1200€	790 (8.9)	10 (6.6)	6 (14.6)	736 (8.7)	38 (13.0)	0.16
	1200–1800€	1882 (21.1)	36 (23.8)	9 (22.0)	1770 (21.0)	67 (22.9)	
	1800–2700€	2479 (27.8)	44 (29.1)	12 (29.3)	2336 (27.7)	87 (29.8)	
	> 2700€	3303 (37.1)	55 (36.4)	11 (26.8)	3147 (37.4)	90 (30.8)	
	Missing data	451 (5.1)	6 (4.0)	3 (7.3)	432 (5.1)	10 (3.4)	
**Marital status, (%)**	Alone	2168 (24.3)	39 (25.8)	13 (31.7)	2016 (23.9)	100 (34.2)	< 0.01
	In a relationship/cohabiting	6737 (75.7)	112 (74.2)	28 (68.3)	6405 (76.1)	192 (65.8)	

^1^Four trajectory profiles for environmental pressure index (EPI) evolution were identified using a latent class model.

The p-values are ANOVA for quantitative variables and chi-square test for the qualitative variables.

**Table 2:** Change over time (2014–2022) of the environmental indicators (NutriNet-Santé study, N = 8,905)^1^.

	**Total**			**Trajectory 1 (Orange)**			**Trajectory 2 (Blue)**			**Trajectory 3 (Green)**			**Trajectory 4 (Purple)**		
	**2018**	**2022**		**2018**	**2022**		**2018**	**2022**		**2018**	**2022**		**2018**	**2022**	
**Crude model**	**(N = 8905)**	**(N = 8905)**	**%**	**(N = 151)**	**(N = 151)**	**%**	**(N = 41)**	**(N = 41)**	**%**	**(N = 8421)**	**(N = 8421)**	**%**	**(N = 292)**	**(N = 292)**	**%**
GHGe	4.11 (2.34)	3.63 (2.10)	−12	6.98 (4.97)	2.40 (1.35)	−66	3.74 (1.75)	9.93 (5.26)	166	4.14 (2.23)	3.70 (2.02)	−11	1.75 (1.02)	1.52 (0.80)	−13
energy use	17.8 (7.30)	16.3 (6.85)	−8	25.0 (12.4)	12.3 (5.93)	−51	16.3 (6.46)	32.5 (11.8)	99	17.9 (7.06)	16.6 (6.67)	−7	10.2 (4.34)	9.14 (3.87)	−10
ecological infrastructures	0.84 (0.51)	0.74 (0.45)	−12	1.47 (1.14)	0.89 (0.72)	−61	0.77 (0.42)	1.92 (1.05)	150	0.84 (0.49)	0.75 (0.44)	−11	0.48 (0.31)	0.44 (0.29)	−8
water use	0.37 (0.19)	0.37 (0.18)	−1	0.48 (0.35)	0.32 (0.15)	−35	0.33 (0.16)	0.50 (0.24)	49	0.36 (0.18)	0.37 (0.18)	1	0.39 (0.22)	0.35 (0.18)	−9
pesticide use	23.7 (11.7)	21.7 (12.1)	−8	30.8 (15.2)	14.1 (10.5)	−54	21.1 (11.4)	44.1 (20.3)	109	24.1 (11.4)	22.2 (11.8)	−8	8.71 (5.86)	6.83 (4.88)	−22
land occupation	10.7 (6.28)	9.50 (5.54)	−11	18.5 (14.3)	6.70 (3.53)	−64	9.82 (4.85)	26.5 (15.6)	170	10.8 (5.97)	9.63 (5.35)	−11	5.48 (2.94)	5.01 (2.39)	−9
EPI	20.3 (9.68)	18.5 (9.22)	−9	30.8 (15.7)	12.1 (7.21)	−61	18.0 (8.21)	42.7 (17.2)	137	20.5 (9.35)	18.8 (8.92)	−8	9.78 (5.34)	8.11 (4.37)	−17
**Adjusted model**															
GHGe				7.01 (0.15)	2.46 (0.13)	−65	3.97 (0.28)	9.50 (0.25)	139	4.33 (0.02)	3.87 (0.02)	−11	2.02 (0.11)	1.76 (0.09)	−13
energy use				24.74 (0.39)	12.24 (0.35)	−51	16.95 (0.75)	30.36 (0.68)	79	18.43 (0.06)	17.00 (0.05)	−8	10.98 (0.28)	9.82 (0.26)	−11
ecological infrastructures				1.47 (0.03)	0.59 (0.03)	−60	0.81 (0.06)	1.82 (0.06)	125	0.87 (0.0047)	0.78 (0.0042)	−11	0.53 (0.02)	0.48 (0.02)	−8
water use				0.46 (0.01)	0.30 (0.01)	−36	0.33 (0.03)	0.44 (0.02)	33	0.36 (0.0019)	0.36 (0.0018)	0	0.39 (0.01)	0.35 (0.01)	−9
pesticide use				31.04 (0.77)	14.55 (0.77)	−53	22.39 (1.47)	41.87 (1.47)	87	25.13 (0.11)	23.20 (0.11)	−8	10.19 (0.55)	8.15 (0.55)	−20
land occupation				18.56 (0.40)	6.84 (0.35)	−61	10.39 (0.77)	25.30 (0.66)	111	11.26 (0.06)	10.05 (0.05)	−8	6.16 (0.29)	5.61 (0.25)	−17
EPI				30.55 (0.50)	12.05 (0.46)	−63	18.99 (0.96)	39.97 (0.88)	144	21.22 (0.07)	19.45 (0.07)	−11	10.96 (0.36)	9.11 (0.33)	−9

Abbreviations: EPI, environmental pressures Index; GHGe, greenhouse gas emissions.

^1^Values are adjusted for energy intake and sex.

### Trajectory profiles and environmental pressures

**Fig. 1:**
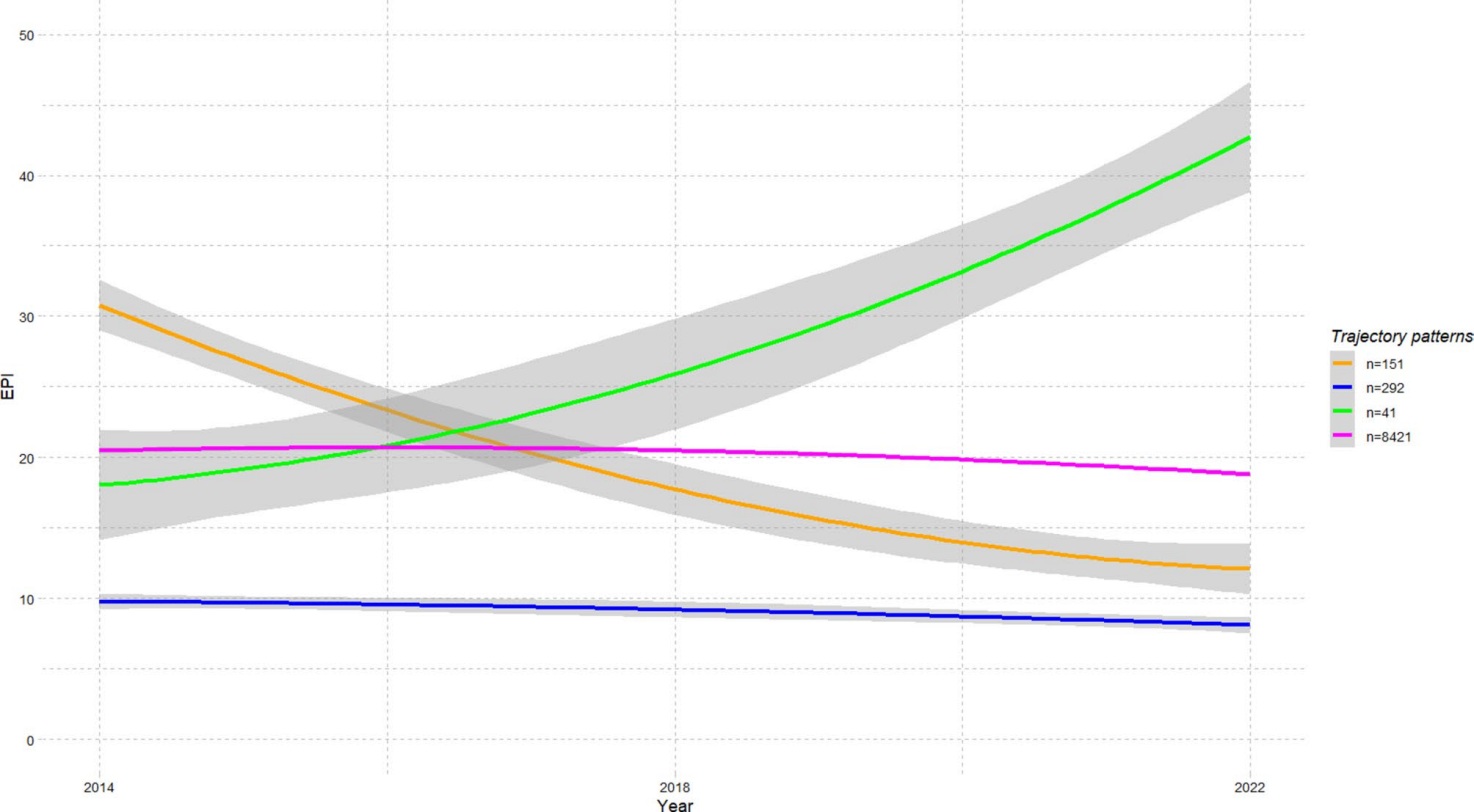
Estimated mean trajectory of summarized environmental pressure index (EPI) within each of the trajectory profiles identified (NutriNet-Santé study FFQ, 2014–2022, N = 8,905). EPI, Environmental pressures index.

Overall, most of the environmental pressures showed improvement over time, ranging from a −1% very slight reduction in water use to a −12% reduction in GHGe. However, the ecological infrastructures values deteriorated over time. The study identified four distinct groups based on their EPI changes over time (Fig. 1 and Table 2). The deterministic starting values using the results of the 1-class model was always the best converged solution (lowest BIC) and nearly all starting values led to the same solution, indicating high model stability. In a sensitivity analysis using time as a categorical variable rather than a continuous one, the resulting trajectories and class memberships were highly consistent with the main analysis. These profiles identified both on their initial level and changes over time will be called trajectory profiles throughout the manuscript. The dominant profile (EPI3, green profile, n = 8421), representing 94.6% of the total population, remained stable over time. This group experienced an 11% reduction in GHGe, and an 8% decrease in pesticide use, although WU remained steady (+ 1%). The second most numerous profile (EPI4, purple profile, n = 292), representing only 3.3% of the population, also showed stable over time, although at lower EPI levels compared to the dominant group. Additionally, one minority profile (EPI2, blue profile, n = 41) showed a significant increase in EPI (+ 137%), while another minority profile (EPI1, orange profile, n = 151) saw an EPI decrease, from a high baseline level to eventually align with the stable low profile, reflecting a −61% change over time. This latter group had average levels of environmental pressures at baseline, but over time, it demonstrated a 66% reduction in GHGe, a 35% decrease in water use, and a 54% reduction in pesticide use. Sensitivity analyses after reclassification of the excluded individuals did not modify the results (data not shown).

Similarly, between two and four trajectories of change were identified for the pressures related to individual indicators **(**Supplemental Fig. 3). However, the identified subgroups differed for each environmental indicator (Chi^2^ < 0.001 for all comparisons, Supplemental Table 2).

The profiles of change in WU over time were notably distinct from those of the other indicators. Indeed, only two profiles could be identified, each one representing an important part of the population (32% and 68%). More precisely, approximately one-third of the population increased (+ 13%) its food production-related water use, and two-thirds decreased (−10%) it.

Two environmental indicators had three profiles, namely LO and pesticide use.

For LO, most of the population (83%) had a medium food production-related LO at baseline (in 2014) which decreased later, 8% of the sample had a high LO at baseline which then increased and 9% had a low LO at baseline which slightly reduced over time.

Regarding pesticide use, one profile was characterized with a stable high pesticide use (78% of the population), one with a stable low pesticide use (11% of the population), and one with an intermediate and reducing pesticide use (11% of the population).

The three other indicators (CED, GHGe, and ecological infrastructures) showed 4 trajectory profiles. Generally, two profiles at different levels (low and medium) remained stable over time, collectively encompassing the majority of the total sample. As for CED and ecological infrastructures, the other two profiles either showed an increase or a decrease over time, with the latter starting at the highest level. The profiles with increasing pressures had low levels for ecological infrastructures and medium levels for energy. Regarding GHGe, two profiles showed decreasing pressures over time, but one of them increased again.

**Fig. 2:**
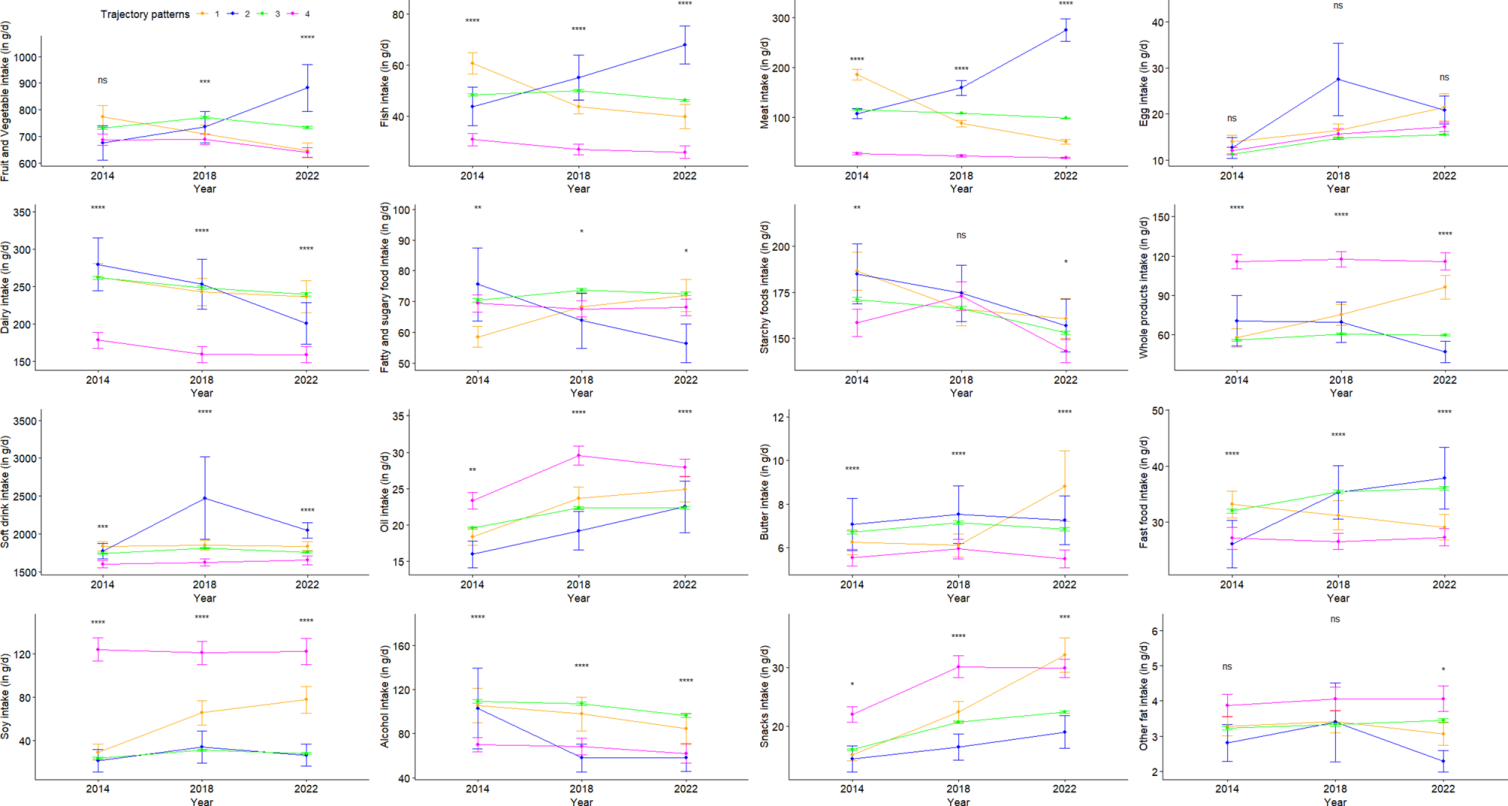
Evolution of the food group consumptions between 2014, 2018, and 2022 across clusters identified using the EPI (NutriNet-Santé study, n = 8,905)^1^. ^1^Four trajectory profiles for EPI evolution were identified using a latent classes model. N is the number of individuals classified in the profile. The mean (95% IC) trajectories are represented with 95% confidence intervals. Food group consumptions are compared using ANOVA. * p-value < 0.05; ** p-value < 0.01; *** p-value < 0.001; **** p-value < 0.0001.

Figure 2 shows the evolution of food consumption for each EPI evolution profile. As expected, the profiles showing an EPI increase or decrease were marked by a strong increase or decrease in meat consumption, respectively, in contrast to dairy product consumption which did not substantially change over time. The profile stable at low EPI levels was characterized by the lowest consumption of animal-based food (meat, fish, and dairy) and high consumption of whole grains products, oil, and snacks. On the opposite, the profile with the highest final level of EPI (at the 2022 time-point) exhibited an increase in red meat consumption.

## Discussion

In the present study, we identified four groups derived from unsupervised trajectories of dietary-related environmental pressures among French adults show that about 95% of the population (EPI3) has stable environmental pressures over an 8-year period. Two other much smaller, groups (EP1 and EPI4; 1.7 and 3.3%) with different starting points showed low final pressure in 2022, but the fourth group (EPI2), with only 0.5% of the population, doubled its diet-related environmental pressures. In the groups with stable pressures, reallocations of consumption between food groups were observed. Interestingly, the group with the lowest baseline pressures (EPI4) remained at the lowest level, and the group with the highest baseline level decreased to reach a low level. The importance of the groups having stable environmental pressures is coherent with previous work on this population^25^ and was also found when analyzing trajectories identified using each environmental indicator separately. It is important to note regression-to-the-mean cannot be eliminated in such analyses^26^. However, it should be limited by having three data points.

Few studies have explored changes over time in the environmental pressures of individual diets. A study by Biesbroek et al. reported a significant decrease in diet-related GHG over a 20-year period from 1993–1997 to 2015 in Denmark^21^. This decrease was measured across the entire food production lifecycle, including waste and losses at all stages. However, when adjusting for energy intake, this reduction vanished, and even increased emissions were observed among men (+ 5%). This suggests that the dietary pattern, the distribution of consumption across different food groups, in 2015 was more carbon-intensive, despite the overall decrease in consumption, likely due to advancing age of the cohort. In contrast, a 12% overall decline in diet-related GHGe was observed in this study. This average decrease varied from a reduction of 66% to an increase of 166% depending on the trajectory group (−65% to + 139% when indicators were adjusted for sex and energy intake). It is important to note that our study covers a more recent period and that participants are likely to be characterized by greater awareness of both the environmental emergency^27^ and its health effects, which could explain, among other things, discrepancies between studies as well as differences in population characteristics. Two studies also documented decreased GHGe over time in recent years^28,29^. Firstly, in the U.S., analysis of representative NHANES repeated surveys shows that the average GHGe associated with the American diet decreased by about 40%, from 4.02 kgCO_2_eq/day to 2.45 kgCO_2_eq/day, between 2003 and 2018. During this period, average energy intake remained stable, but the significant reduction in GHGe was mainly due to a 40% decline in per capita beef consumption^28^. Then, a study based on the food balance sheet showed that between 1986 and 2017, GHGe from the United Kingdom ‘s food supply decreased by 20%, with per capita emissions falling by 32%. According to the authors of the study, this reduction was mainly driven by changes in supply rather than demand. When comparing different food groups, it was noted that the decreased supply of beef, lamb, and pork significantly contributed to this overall reduction^29^.

Research exploring the relationships between environmental pressure indicators and dietary choices is becoming more common in the scientific literature^30–33^. Among the various environmental metrics examined, GHGe and LO are the most frequently studied indicators and are highly correlated^31,32^. Numerous population-based observational studies have consistently shown that diets primarily based on plant products produce significantly lower GHGe and require less land than meat-inclusive diets. In particular, beef, pork and processed meat, along with dairy products to a lesser extent, are associated with the highest environmental impacts across various indicators, including GHGe, land and water use, acidification and eutrophication^34–36^. Despite the varying degrees of reduction, this explains the reduction in GHGe in most trajectories. For example, trajectory EPI1 with the highest decrease in EPI score is characterized by a decrease over 8 years of all types of meat (beef, pork, poultry, and offal). In contrast, the trajectory that indicates an increase in GHGe (EPI2) over time consists of participants whose consumption of various types of meat, especially beef and poultry, rose across the study period. Of note, this last group was very small (n = 41), corresponding to 0.5% of the analyzed sample, and the increase in GHGe is accompanied by an increase in water use and density of ecological infrastructures. This aligns with scientific knowledge about food groups that require significant water, including cereals and fruits, with some discrepancies noted when considering total blue or green water^37,38^. As a factor of ecosystemic services, ecological infrastructures indicator can be seen as a proxy for functional biodiversity. Extensive farming systems generate the most ecological infrastructures per kilo of food product. Therefore, ruminants raised on grasslands (for milk and meat) are the main contributors to biodiversity related to agriculture. While pastures provide important ecosystem services and functional biodiversity^39^, livestock farming is a major factor of deforestation, which is detrimental to wild biodiversity^34^.

Overall, the group with the lowest and consistently low EPI was underrepresented in this population, comprising only about 3.3% (n = 292). These individuals tended to have a low intake of animal products and consume a significant amount of organic foods. This may reflect the eating habits of committed populations who maintain these habits over time, compared with the trajectory (1.7%, EPI1) which increases the environmental pressures associated with their food consumption. Conversely, the group that strongly increased the EPI may illustrate some segments of the population with difficulties in maintaining sustainability over time possibly due to economic issues, as these participants often have low levels of education, low levels of monthly income, or are unemployed.

This study was conducted within the French context and is based on a substantial cohort providing longitudinal data on diet through the same data collection instrument, differentiating between modes of production. However, the applicability to other nations, particularly those in Western regions, remains uncertain, given that dietary customs, responsiveness to the crucial transition towards sustainable consumption, and agricultural methodologies may vary. Consequently, it would be advantageous for this approach to be replicated in other settings contexts.

While the study was based on a population voluntarily recruited to participate in a nutrition and health research, and thus, which has been shown to exhibit more sustainable and healthier behaviors than the general population^27^, the decrease in environmental pressures appeared to be rather low, if compared with what might have been expected to keep food within planetary limits^40^. Furthermore, certain segments of our study’s sample still exhibit significant food-related environmental pressures, and some individuals, though very few in proportion, even increase their diet-related environmental footprints. These findings raise doubts about public policies’ effectiveness so far in encouraging the population to adopt more environmentally-friendly diets and maintain these changes over time. Our findings align with previous work in which we reported that certain sub-groups of the population resist changing their dietary profiles, especially regarding animal products^41,42^, which may be explained by sociocultural barriers^43^. This issue must be viewed in light of the French public’s stance on environmental policies, which necessitates targeted communication campaigns^44,45^.

The strengths of the study encompass that we distinguished between organic and conventional farming methods to more accurately evaluate the impacts of different dietary patterns. Our study utilized various environmental indicators, including some that have received less attention, such as water use, pesticide use, and ecological infrastructures that reflect biodiversity. To the best of our knowledge, this is the first study which includes all these indicators while considering two different types of farming. It is however important to acknowledge the limitations of the water use indicator concerning different farming systems. Thus, in this study, it was assumed that the percentage of irrigated area and the water use per hectare were identical for both organic and conventional farming. However, this simplification may not accurately reflect actual differences. Therefore, further research is needed to better characterize irrigation practices in organic and conventional farming. Finally, our sample of nearly 9,000 individuals enabled us to capture a variety of dietary habits and track changes in diet over a significant recent period, during which awareness of sustainable diets may have increased. Some limitations of our study should be highlighted. First, our findings are based on a sample of volunteers participating in a long-term cohort focused on nutrition and health. Therefore, caution is necessary when generalizing these results to the broader population^46^, as not all behaviors may be represented. The respondents were predominantly women, older participants, postgraduates and more healthier lifestyles on average, compared to the general population. It is however difficult to limit this sampling of volunteers as longitudinal studies need a strong implication and thus select a specific population. Second, our evaluation of environmental impacts was limited to the production step and did not encompass downstream stages such as processing, transformation, packaging, and transport. This restriction may understate the overall environmental impacts, particularly for highly processed or imported products, but they typically occur mainly at the production stage^47^. Therefore, it is essential to conduct comprehensive farm-to-grave life cycle analyses that encompass the entire supply chain and account the diversity of agricultural practices.

In an era where food systems account for nearly one third of global GHGe and contribute significantly to environmental degradation, this study provides a unique perspective by analyzing long-term environmental pressures related to changes in dietary patterns among a large sample of French adults. Using an extensive set of environmental indicators, including novel metrics like ecological infrastructures and pesticide use, our findings reveal that most participants in this specific cohort show a moderate decrease in dietary-related environmental pressures over time, with only a small fraction showing substantial changes. Our results show that while awareness of sustainability may be increasing, it has yet to be translated into significant behavioral shifts for most individuals. This highlight the pivotal role that targeted policies can play in driving sustainable changes, particularly among groups who struggle with maintaining environmentally friendly diets due to social or economic barriers.

## Methods

### Study population

The current study is based on longitudinal observational data from 2014 to 2022, using a sub-sample of the NutriNet-Santé study. The NutriNet-Santé study is a prospective ongoing cohort conducted in French volunteer adults with internet access. Informed consent was obtained from all individual participants included in the study. Consent covered both participation in the research and the publication of the results. Since 2009, this online-based cohort has examined the factors influencing diets, nutritional status, lifestyles, and their links with health outcomes^48^. Participants are required to complete annual or biannual questionnaires to collect this information, and additional questionnaires are periodically administered. Sex, occupational status, income, place of residence, physical activity levels (using the International Physical Activity Questionnaire (IPAQ)^49^, anthropometric data, and smoking habits are all self-reported using validated questionnaires^50–52^.

The NutriNet-Santé study was conducted in accordance with the principles of the Declaration of Helsinki. The study protocol was reviewed and approved by the Institutional Review Board of the French Institute for Health and Medical Research (INSERM) through its Ethical Evaluation Committee (CEEI; reference no. 0000388FWA00005831). Approval was also obtained from the French National Commission on Informatics and Liberty (CNIL; approval nos. 908450 and 909,216). Furthermore, the study is registered on ClinicalTrials.gov (Identifier: NCT03335644).

### Data collection

#### Dietary data

In 2014, 2018, and 2022, food consumption data were collected using an Organic Food Frequency Questionnaire (Org-FFQ), assessing the consumption, over the 12 past months, of 264 food and beverage items, as previously described^27,53^. Participants were asked to report the consumption frequency for each food and beverage item, regarding the production method (conventional vs. organic). The portion size of each item consumed was reported using unit portions (i.e., a slice of bread, a yogurt of 125 g, an egg), accurate quantities (i.e., a bowl, a glass, a teaspoon), or photographs that allowed a visual estimation of the quantity consumed. Each item’s consumption was then converted into grams per day. For each food item of the Org-FFQ, the frequency of organic consumption was reported using a Likert scale: always, often, about half of time, rarely, or never. Then, to obtain a consumption in g/d, this scale was translated into 100, 75, 50, 25, and 0% of the total food item consumption. The overall organic consumption was, therefore, the sum of these consumptions^27^.

#### Sociodemographic data and other covariates

Sociodemographic variables at baseline and yearly after were self-reported by each participant, including sex, age, education (< School diploma, High school diploma, and Post-secondary graduate), tobacco status (non-smoker, former smoker, and current smoker), socio-professional category (“Retired”, “Executive or higher intellectual profession”, “Craftsman, trader, business manager, farmer”, “Intermediate occupation”, “Employee”, “Unemployed”, “Others without professional activity”, “Student”, and “Manual Worker”), residential area (Rural area, Urban area < 20,000 inhabitants, Urban area 20,000—200,000 inhabitants, Urban area > 200,000 inhabitants), physical activity level according to the IPAQ (High, Moderate, Low, Missing data)^54^, monthly household income per unit of consumption (< 1200€, 1200–1800€, 1800–2700€, > 2700€, unwilling to declare), and marital status (Alone, In a relationship/cohabiting). Body mass index (BMI) was calculated based on self-reported weight and height data^50–52^.

#### Environmental pressures

Food consumptions were merged with their corresponding environmental value. We considered six indicators, namely GHGe, cumulative energy demand (CED), land occupation (LO, ecological infrastructures, pesticide use estimated by the treatment frequency index, and water use (WU). The method to compute GHGe, CED, and LO has been extensively described elsewhere^55^. Briefly, farm-to-farm life cycle assessments (LCA) from the DIALECTE database developed by Solagro^56^ were used to compute food-related environmental indicators distinguishing organic and conventional farming methods. This database specifically covers conventional and organic French farms. GHGe (the indicator calculated in this study is the Global Warming Potential (GWP) over a 100-year time horizon (GWP_100_) in kg of CO_2_ equivalents (CO_2_eq)), CED (MJ), and LO (m^2^) were computed at the farm perimeter excluding downstream steps such as conditioning, transport, processing, storage or recycling stages. Economic allocation (accounting for coproducts), as well as cooking and edibility coefficients, were applied to 92 raw agricultural products to estimate production environmental pressure for the 264 FFQ food items, each displayed into their organic and conventional forms. Extensive details have been provided previously^57^ and are provided in Supplemental Method 1.

Ecological infrastructures, pesticide use and WU indicators were used for the first time. These have been computed for both organic and conventional foods using different databases, in particular the French annual agricultural statistics, FAOSTAT, Surveys on farming practices in France (Agreste website, Agriculture minister), Agribalyse, Graphic parcel register, BD Haie, BD Forêt®, effectives wetlands. A detailed description of the computation of the three newly developed indicators is provided in Supplemental Method 2.

For each indicator, a higher value reflects greater pressure, except for ecological infrastructures reflecting preservation of biodiversity. Based on the six environmental indicators, a summary of the environmental pressure index (EPI) was computed (in reverse order for ecological infrastructures). In detail, each indicator was first standardized to a scale of 0 to 1 to ensure comparability across variables and remove differences in magnitude or units. For agroecological infrastructures, a high value is considered positive; therefore, the result was subtracted from one. The six standardized indicators were then summed with equal weighting and rescaled to stay within the same range of 0 to 1. The final sum was then multiplied by 100 to produce an EPI that ranges from 0 to 100. A higher EPI indicates a greater environmental impact. The distribution of the EPI is shown in the Supplemental Method 3.

### Statistical analysis

A total of 9,095 participants who had data on food consumption for each collection period (2014, 2018, and 2022) were eligible for this study. Individuals classified as underreporting or overreporting their energy intake or living overseas were excluded, as detailed in a previous publication^27^. Individuals with more than 1,500 kcal variation between 2014 and 2022 (corresponding to the 1% with the highest kcal increase or decrease) were considered outliers and also excluded (n = 190), leaving a population for the trajectory profile analyses of 8,905 (flowchart in Supplemental Fig. 1), for whom models were performed.

The trajectory profiles were identified using latent class modeling for each six environmental pressures (GHGe, CED, WU, LO, pesticide use, and ecological infrastructures) and the EPI using the R package lcmm^30^ adjusted for age, sex, and energy intake. The models included random intercepts and timepoints were treated as continuous. The “beta” link function, belonging to the family of Beta cumulative distribution functions, was employed to estimate the relationship. Models with one to six trajectory profiles were tested, and those failing to converge after 5,000 iterations were excluded. To guide class enumeration, we relied primarily on the Bayesian Information Criterion (BIC), and also examined AIC, SABIC, entropy, and ICL, which yielded consistent class solutions. Because LCMMs are sensitive to local maxima, we implemented a multi-start procedure using the 1-class model as deterministic starting values together with nine additional random initial values for each model with ng ≥ 2. The best converged solution (lowest BIC) among the 10 runs was retained. Log-likelihood trajectories, parameter estimates, and posterior classification quality were examined to ensure robust convergence. As a sensitivity analysis, we also re-estimated the models using categorical rather than continuous time.

The profiles identified with the different environmental pressure variables were compared using χ^2^ tests and the strength of each association was examined through a Cramer’s V test. The identified profiles were then compared for sociodemographic data at baseline and food group consumption (n = 25) using ANOVA or mixed models. P < 0.05 was considered statistically significant.

In sensitivity analyses, the 190 excluded individuals were reclassified into the respective profiles, after which the profiles were compared based on environmental pressure and sociodemographic characteristics. Additionally, among participants who completed the FFQ in 2014, excluded and included individuals were compared. R Software (version 4.3.2, R Foundation for Statistical Computing, Vienna, Austria^58^) and SAS Software ® (version 9.4, SAS Institute INC, Cary, NC, USA) were used for statistical analyses.

## Transparency

Dr Kesse-Guyot (the guarantor) affirms that the manuscript is an honest, accurate, and transparent account of the study being reported, that no important aspects of the study have been omitted and that any discrepancies from the study as planned have been explained.

## Supplementary Information

Supplementary Information.

## Data Availability

Analytic code will be made available upon request pending. Researchers from public institutions can submit a collaboration request, including information on the institution and a brief description of the project, to the following point of contact: Email: [collaboration@etude-nutrinet-sante.fr](mailto:collaboration@etude-nutrinet-sante.fr) All requests will be reviewed by the steering committee of the NutriNet-Santé study. If the collaboration is accepted, a data access agreement will be necessary and appropriate authorisations from the competent administrative authorities may be needed. In accordance with existing regulations, no personal identifying data will be accessible. The corresponding authors may also be contacted, and they will guide you to the appropriate procedure.

## Abbreviations

BIC: Bayesian information criterion
CED: Cumulative energy demand
EPI: Environmental pressure indicator
FFQ: Food frequency questionnaire
GHGe: Greenhouse gases emissions
IPAQ: International physical activity questionnaires
LCA: Life cycle assessments
LO: Land occupation
WU: Water use
