# Supplementary material for: Exploring eight-year trajectories of diet-related environmental pressures in the NutriNet-Santé cohort
Source: Sci Rep. 2025 Dec 6;16:412. doi: 10.1038/s41598-025-29786-6 (PMC12770496; doi:10.1038/s41598-025-29786-6)
Supplement: Supplementary file 1 — Supplementary Information. [file 41598_2025_29786_MOESM1_ESM.docx]

Supplemental Material: Exploring eight-year trajectories of diet-related environmental pressures: Results from the NutriNet-Santé cohort.

Perraud et al.

Table des matières

[Supplemental Method 1: conversion from raw agricultural products to food items 2](#_Toc204673746)

[Supplemental Method 2: methodology used for the assessment of the three newly developed environmental indicators: pesticides use, water use, and ecological infrastructures 3](#_Toc204673747)

[Supplemental Method 3: Description of the environmental pressures index (EPI) 9](#_Toc204673748)

[Supplemental Table 1: Comparison of participants in the BioNutriNet Project with and without 3 dietary evaluations^1^: 10](#_Toc204673749)

[^1^ Values are mean (SD) or %, p referred to t-test or Chi² test 10](#_Toc204673750)

[Supplemental Table 2: Number of individuals for each profile identified for each environmental pressure studied across profiles of EPI 12](#_Toc204673751)

[Supplemental Figure 1: Participants’ selection flowchart based on the three food frequency questionnaires from the NutriNet-Santé cohort. 13](#_Toc204673752)

[Supplemental Figure 2: Correlation between environmental indicators (pressures/d) 14](#_Toc204673753)

[Supplemental Figure 3: Specific trajectories for each individual environmental indicator (NutriNet-Santé study FFQ, 2014-2022, N = 8,905)^1^ 14](#_Toc204673754)

# Supplemental Method 1: conversion from raw agricultural products to food items

As our goal was to assess the environmental impacts of diets, we conducted a set of conversions to obtain data at the consumer level.

Conversion from raw agricultural products to ingredients

As a first step, to obtain values for each indicator and ingredient from agricultural raw products, economic attributions by co-products were applied followed by the allocations of mass, cooking, and edibility coefficients in order to obtain indicator values for the ingredient as consumed, using data from literature and NutriNet-santé composition database (for the allocations of cooking and edibility coefficients). The same conversion factors were used for organic and conventional products.

Data were unavailable for 59 minor ingredients which accounted for more than 5% in at least one recipe (*e.g.* tropical areas, certain alcoholic beverages, grains or yeast). Their environmental impacts were considered as null.

Conversion from ingredients to food items

Each of the 264 items of the Org-FFQ (Organic Food Frequency Questionnaire) was composed of sub-items (or foods). For example, the item pasta was composed of many sub-items (vermicelli, pasta, Chinese noodles, egg pasta, fresh pasta, and egg and fresh pasta spinach flavor). The composition of each item was the result of the multiplication of gender-specific consumption frequency of each sub-item (determined using the 24-h dietary record tool of NutriNet-Santé) by the ingredients constituting the sub-item.

Overall, a total of 766 ingredients are components of the 264 food items of the Org-FFQ. For feasibility reasons, the environmental impacts of the ingredients which accounted for at least 5% of the recipe were computed, corresponding to 442 ingredients.

About 75% of food items had more than 98.8% of their composition covered by the 442 ingredients. When an ingredient was missing for a given item, environmental impacts were ‘standardized’ for 100g. The ingredients especially concerned by the standardization were fish products, jam, cheeses and dressings.

Finally, we obtained environmental impacts for most of the 264 food items in organic and conventional forms. No data were available for 22 items for CED, 21 items for GHGE and 25 items for land occupation. Missing data included the following food items: water, certain alcoholic beverages, tropical fruit or vegetables, grains and certain oils. We opted not to assign average category level values as no reliable proxy exists at the food level. Thus, imputing values from average category may disort results rather than improve accuracy.

# Supplemental Method 2: methodology used for the assessment of the three newly developed environmental indicators: pesticides use, water use, and ecological infrastructures

Methodological choices and assumptions:

The newly developed environmental indicators have been calculated for 84 agricultural products, including 73 plant products and 11 animal products. Indicators for fishery and aquaculture products have not been calculated.

Two farming systems were considered: "conventional" (i.e., non-organic) and "organic" agricultural methods as defined in European Commission (EU) 2018/848^1^.

For products produced in France, French references are used, while foreign references are used for imported products. The term "organic agriculture" refers to production methods that meet the standards set out in the European Union’s régulations1. All other production methods that do not meet these standards are classified as "conventional".

We ensured that the sources correspond to the geographical areas where the food consumed in France was produced. The FAO trade matrices^2^ are used to identify the main countries producing and exporting to the European Union and France. The supply balances were compiled using the MOSUT^3^ tool designed by SOLAGRO, based on data from the supply balances^4^ between 2017 and 2020. These assessments were then used to categorize products into two groups: those that are "mainly imported" (imports/resources > 50%) and those that are "mainly produced" in France (imports/resources < 50%). In total, references for 12 products produced outside France were sought: Coffee, Cocoa, Tea, Orange, Grapefruit, Lemon, Rice, Olive, Walnut, Green Bean, Soy, and Tomato.

For each product defined as “mainly imported”, the main producing countries were identified based on production and trade data available in FAOSTAT^5^. In the case where data on production yields, pesticide use, and water consumption (mostly irrigation) were available for only one of the main producing countries, that data was used for calculation. For example, soya flour used in animal feed is mainly imported from Brazil in conventional farming and from Togo, India, or Ukraine in organic farming^2^. For certain products such as rice, walnuts, and green beans, no references were available for the main producing and exporting countries to France, so French references were used by default.

In 2021, organic farming, accounting for 10.5% of French agricultural land, was included in the statistical data without distinction between conventional and organic methods. Therefore, the average production from the annual statistics was assumed to represent conventional production. Thus, the average yield in "conventional" agriculture was calculated by dividing the total quantity produced by the total cultivated area. Average organic yields were calculated using yield loss coefficients from Dialecte^6^, Agribalyse or scientific publications. For imported products and buckwheat, the FAO average yield was used as yield for conventional farming.

To quantify the environmental indicators for animal products, the land used to produce their feed was considered. The Agribalyse ® database 3.1^7^ provides information on animal food products consumed in France. It uses livestock feed data and regional yields to calculate indicators for products like milk, eggs, and meat. A biophysical allocation method was applied to allocate resources to co-products^8^. Although organic systems for turkey, duck, rabbit, goat's milk, and sheep's milk were not considered in this study, case studies have been adapted for these types of farms.

Computation of the indicators:

***Pesticide footprint***

The plant health treatment frequency index (TFI) is a standardized indicator that measures the frequency of pesticide use for a given crop. The TFI, derived from farmers’ reported practices, was adapted from the Danish indicator^9^. It is defined as the number of reference doses applied per spatial unit over a specified period. In most cases, the spatial unit is the plot, with the period being the crop year. This indicator can then be aggregated at different spatial and temporal scales. Furthermore, the index can be segmented by family or type of plant protection product, by type of treatment, or by type of crop. It can also be broken down into different segments, according to the type of product used: herbicide, insecticide, fungicide, seed treatment, biological control, or other. By aggregating substances with different modes of action, the TFI provides a comprehensive measure of overall pesticide use.

For further details on the French standardized calculation of the TFI, see to the Ministry of Agriculture and Food's methodological guide^10^. In this study, we assessed three TFI: total TFI (excluding biological control products), herbicide TFI and non-herbicide TFI (excluding biological control products).

Biological control products were excluded from the analysis, as our primary focus is on the environmental impact of synthetic pesticides, which generally pose a greater environmental risk compared to biological alternatives^11,12^.

The pesticide footprint quantifies the land area treated with pesticides to produce 1 kg of a given commodity. The calculation method differs for plant and animal products:

- For crops, the footprint is obtained by multiplying the average Total TFI by the inverse of the crop yield.
- For animal products, we first determined the average area of land required to produce 1 kg of product per crop type. This area was then multiplied by the corresponding TFI and multiplied by the inverse of their yield.

The result is expressed as pesticide-impacted area equivalents, referred to as pesticide use. The area impacted by pesticide use is referred to as the pesticide footprint. This includes the herbicide footprint use and the non-herbicide use.

The data used for the computation of pesticides footprint are summarized below (Table 1).

Table 1 Data sources for the computation of the pesticide’s footprint

|  | Data sources |
| --- | --- |
| TFI herbicides, excluding herbicides et total (excluding biological control), conventional | 1. - French surveys on plant protection practices (2017 for field crops, 2018 for fruit growing and vegetables, 2019 for vine growing) 2. -Technical documents and scientific literature 3. -Agribalyse ® 4. -Surveys on the use of plant protection products in Spain |
| TFI herbicides, excluding herbicides et total (excluding biological control) organic | - French surveys on phytosanitary practices (for fruit growing, 2019 for vine growing)   1. -Technical documents from the DEPHY networks |
| Average conventional yield | 1. -Average yield between 2017 and 2021 from annual agricultural statistics (assimilated to average conventional yield) |
| Average organic yield = average conventional yield - (average conventional yield organic yield loss coefficient) | -Organic yield loss coefficient: 2022 arable land French survey  -Technical documents and scientific literature (including BioNutriNet study)  -Agribalyse |

Water use

The Water indicator groups two indicators to characterize agricultural production:

- Water requirements for crop production (irrigation);

- Water requirements for livestock production (watering, and cleaning of facilities).

Several methods have been developed to assess water footprint in recent years^13–15^:

- Pfister et al.^16^( "Withdrawal to Availability" method). It considers both **water consumed (EC)** and **water returned (ER)** to the environment, treating returned water as part of the overall water footprint.

- Hoekstra et al. ^15,17^ (”Consumption to Availability” methods). This method excludes water withdrawals and returns from the calculation, focusing only on water consumption. means that the quantities of water withdrawn and returned to the system (ER) are excluded from the calculation.

- AWaRe method^18^ (Available Water Remaining). This method developed as part of the latest generation of water footprint assessments, calculates water consumed (EC) relative to the water available in the region studied.

Our objective was to quantify the total water withdrawn for food production, rather than just the water consumed by plants. Although some of the withdrawn water returns to the system, water withdrawal represents the volume of water temporarily unavailable for other uses, creating potential competition with other sectors. Therefore, irrigation water used was estimated using the "withdrawal to availability" calculation method developed by Pfister et.al ^13^.

Irrigation water use is significant: in France, representing nearly 3 billion m^3^ per year, including 1 billion m^3^ for maize irrigation and 306 million m^3^ for soft wheat irrigation^19^. The irrigation water indicator highlights the pressure on a product's water resources as a function of its production method (organic and conventional) and practice (m^3^/ha).

The indicator is calculated using the total amount of irrigation water used in mainland France for the crop under study, divided by its total production.

$$water for irrigation\left( \frac{m^{3}}{\mathrm{kg}}of product \right)= \frac{total quantity of water withdrawn}{total production per crop}$$

Total quantity of water used for irrigation is determined by multiplying the irrigated area of the crop in question by the quantity of irrigation applied per hectare:

$${total quantity of water withdrawn}_{region}={irrigated area}_{region} \times{quantity of irrigation per ha}_{region}$$

The irrigated area data are sourced from the Agricultural Census (AC) available on the Agreste website^20^. The most recent available data (2020) were used, as they best reflect current irrigation practices and average climatic conditions. The data were analyzed by region and by crop.

Due to lack of data, to calculate irrigation water usage, it was assumed that the percentage of irrigated area and the amount of water per hectare is the same for both organic and conventional farming. This assumption was necessary because comparative data on organic vs. conventional irrigation practices are scarce. Moreover, irrigation water management depends on various factors as: irrigation technologies (sprinklers, drippers, etc.), soil textures (sandy, loamy, etc.), organic matter percentage, soil preparation, etc.^21^. While irrigation needs may differ between organic and conventional systems, the available data did not allow for a precise differentiation. The only factor we were able to account for was climate, using regional irrigation data^22^. The water use per kg of product is influenced by both yield variation and geographical distribution of production. For example:

- 39% of conventional and 34% of organic maize is cultivated in “Nouvelle Aquitaine” region where the water amount is 199 mm/ha whereas,
- 10% of conventional and 21% of organic maize is cultivated in “Pays de la Loire” region where the water amount is 111 mm/ha.

Although total water use per ha for maize in France is greater for conventional than organic, the yield difference ( ̴30% lower for organic maize) results in higher water use per kg of organic maize.

To estimate the organic irrigated areas, the total irrigated area per region has been multiplied by the proportion of organic farmland in that region.

Irrigation water data (mm/ha) are not systematically available for all crop types in all region. Then data were available from cropping surveys^22^, They were used directly. For the missing data, additional sources were used and validated by experts.

Water indicator for livestock farming (excluding irrigation) was calculated using data from Agribalyse 3.1®, which provides estimates of water used for watering and facility cleaning per liter of milk or kg of meat. The calculation follows the ReCiPe 2016 Midpoint (H) method: Water consumption - market for tap water^23^.

Ecological infrastructure (EI)

EI refers to landscape features that support biodiversity and ecosystem services. These features can be classified into several types:

- Linear or surface tree formations (hedges, copses, trees, agroforestry, etc.),

- Grassed areas (extensive grassland, areas under environmental cover, etc.),

- Cultivated areas (environmental set-aside, extensive arable strips, etc.),

- Ruderal areas (low walls, terraces, grassed paths),

- Wetlands (ponds, springs, wet ditches).

To develop the EI indicator, we standardized all features using a common characteristic variable that could be linked to food or fodder production areas. To ensure robustness and comprehensive coverage, the following features were included:

- Surface area of hedges and linear tree elements

- Surface area of grassed strips (buffer strips along watercourses)

- Surface area of forest edges resulting from an intersection between the BD Forêt® and the GPR (Graphic parcel register)

- Surface area of copses

- Surface area of wet meadows (share of wetlands in permanent pasture by livestock production area)

- Surface of grazed woodland (share of grazed woodland in permanent pasture by livestock production area)

- Surface of fallow land (> 5 years old) (code J6S in GPR 2021)

- Surface of dry-stone walls

- Surface of ponds

Each of these EI was identified using spatial data and quantified in terms of surface area, either by characterizing the surface area directly (wet grasslands, for example) or by multiplying it by an effect coefficient applied to the linear length of the EI.

Priority was given to applying coefficients derived from the CAP11 Ecological Interest Areas^24^.

Grassed strips and fallow lands were assigned to crops in proportion to the length of intersection with the adjacent plots.

Wet meadows and grazed woodland were only assigned to livestock production.

We did not assign any ecological infrastructure to the imported products.

Table 2 Ecological infrastructures data source and unit

| Type | unit | Data source | Used coefficient |
| --- | --- | --- | --- |
| Hedges | Linear meter | Intersection between the plots of the 2021 GPR (Graphic Parcel Register) and the "hedges" layer of the BD TOPO (IGN). | 1 m = 20 m² |
| Grass strips | Square meter | Plots of the 2021 GPR coded BTA | Real surface area |
| Woodland edge (excluding poplar groves) | Linear meter | Intersection between the plots of the 2021 GPR and the linearized BD FORET (IGN) layer | 1 m = 8 m² |
| Wet meadows | Square meter | Intersection between the plots of permanent pastures coded PPH, SPH, SPL, BOP, CAE, CEE in the 2021 GPR and the inventory of effective wetlands from the SIG Wetlands Network: https://sig.reseau-zones-humides.org/ | Actual surface area m^2^ inventoried as "wet" per m^2^ of permanent pastureland |
| Fallow land over 5 years old | Square meter | Plots of the 2021 GPR coded J6S | Actual area m² fallow per m² adjacent crop |
| Grazed woods | Square meter | Plots of the 2021 GPR coded BOP | Actual surface area m² of woodland grazed per m² of permanent pasture |
| Groves | Square meter | Intersection between the plots of the 2021 GPR and the "Zone de vegetation" layer of the BD TOPO (IGN), where the "nature" field is equal to "Bois" | 1 m^2^ = 1,5 m² |
| Dry-stone walls | Linear meter | Intersection between the plots of the 2021 GPR and the "Construction linéaire” layer of the BD TOPO (IGN), where the "nature_detaillee" field is equal to "Mur de pierres sèches" | 1 linear meter = 1 m² |
| Seas | Square meter | Intersection between the plots of the 2021 GPR and the "Plan d’eau" layer of the BD TOPO (IGN), where the "nature" field is equal to "Mare" | 1 m^2^ = 1,5 m² |

Abbreviations: GPR, graphic parcel register ; BD TOPO IGN, Institut national de l'information géographique et forestière topographic database

Comparison with national figures

To validate the results obtained, the calculated indicators per kilo of raw product were multiplied by the quantities produced in mainland France or by the quantities imported (for soy) and compared to national data.

▶In 2024, the total pesticide footprint of the plant products considered in conventional agriculture is estimated at 57.7 million hectares in France ^25^. As part of the ADONIS project, Solagro used the same calculation method (based on TFI) to assess the pesticide use frequency at the municipal level. The sum of the ADONIS TFIs for mainland France is 60.1 million hectares. The difference can be explained by the fact that the considered products do not cover all treated crops (e.g., seed production is excluded). Additionally, the 57.7 million hectares estimate does not include organic farming, or feed production for livestock. Despite these limitations, the results align closely with national estimates, validating the order of magnitude of the calculated pesticide indicators.

▶The total annual irrigation water use for the considered products is 2.7 billion m^3^.

According to the “Banque nationale des prélèvements quantitatifs en eau”^19^, the volume of water withdrawn for irrigation in France was 3.1 billion m^3^/year between 2017 and 2020. Since the considered products account for 85% of irrigated land, this confirms the validity of calculated irrigation water indicator. Using the "Water consumption" indicator from the Agribalyse ReCiPe 2016 Midpoint (H) method, water use for livestock watering and cleaning buildings is estimated at 234 million m^3^.

There are few recent references on overall water use. A 2001 study by the French Institute for the Environment estimated water consumption at ~400 million m³^26^.

However, since 2001, the number of cattle and pigs has fallen. IDELE (Institut de l’élevage, French livestock institute) now estimates that “*the water footprint of dairy and meat products is of the order of 1 to 3 liters of water per liter of milk and 30 to 50 liters of water per kilo of live meat (at the farm gate)*”, which confirms the order of magnitude used in in this project, but with a higher footprint for milk (6L for 1L of milk) and a lower footprint for meat (27L for 1kg of meat)^27^. These estimates require further consolidation.

According to these figures, nearly 60% of the watering and washing water footprint is accounted for by dairy cattle, 15% by beef cattle and 11% by pigs.

▶To validate the EI, we applied the coefficients calculated for each EI to national agricultural production in mainland France. The expected results should correspond to the length or surface area of the EI in France (excluding areas that are not considered int the perimeter, such as seed production, sorghum, etc.).

The obtained values are higher than those of the source data, which come from the intersection between the plots in the GPR and the EI layer. This discrepancy arises because the GPR covers only 80-85% of cultivated areas in France. Therefore, EI coverage is likely higher than those intersected by the GPR. Wet grassland extrapolated from animal products covers 302,638 ha, compared to an identified total of 328,574 ha, representing 92% agreement.

Grazed woodland extrapolated from animal products covers 229,537 ha, compared to an identified total of 290,048 ha, representing 79% agreement.

# Supplemental Method 3: Distribution of the environmental pressures index (EPI)

A synthetic Indicator of Environmental Pressures (EPI) was calculated by normalizing each indicator to a scale of 0 to 1. For agroecological infrastructures, a high value is considered positive; therefore, the result was subtracted from one. These standardized values were then summed and rescaled to stay within the same range of 0 to 1. The final sum was then multiplied by 100 to produce an EPI that ranges from 0 to 100. A higher EPI indicates a greater environmental impact. Although no explicit weight is assigned to the indicators in the calculation of the Environmental Pressure Index (EPI), standardisation introduces an implicit weight related to their observed variability. The most variable indicators, such as greenhouse gas emissions, therefore have a greater influence on the EPI than those less variable. The index thus becomes more sensitive to indicators with high fluctuations, reflecting their empirical distribution rather than an arbitrary weighting choice. The distribution of the EPI is showed below:


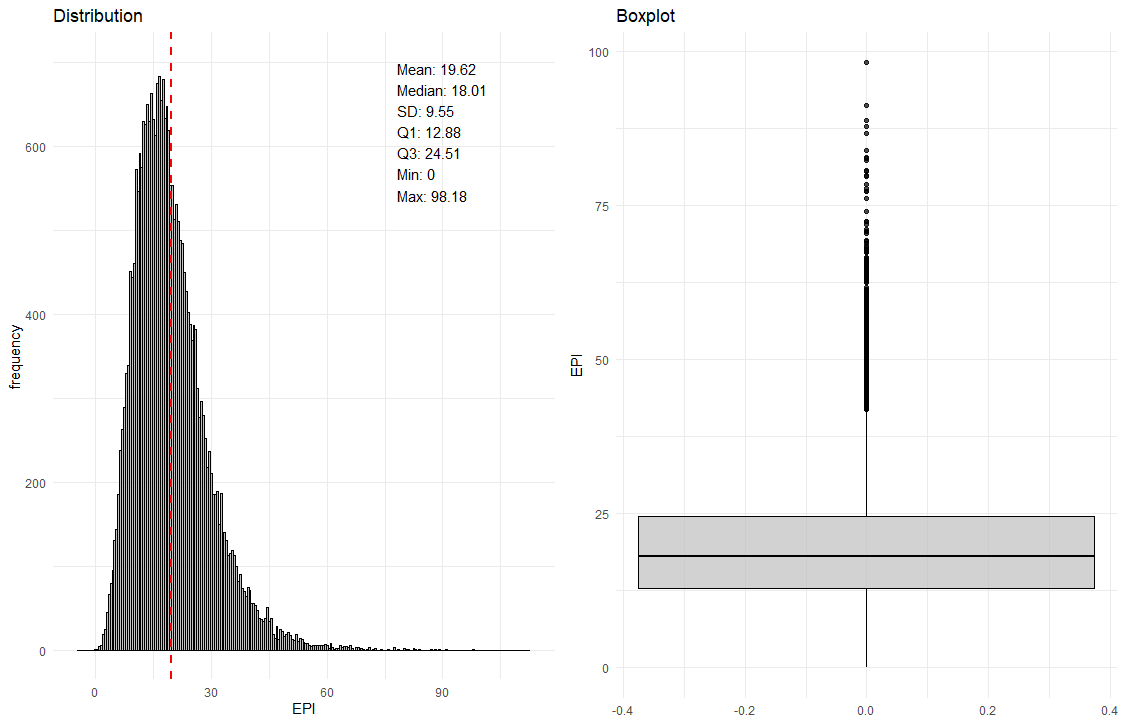


The correlation between EPI and individual environmental indicators are shown below:
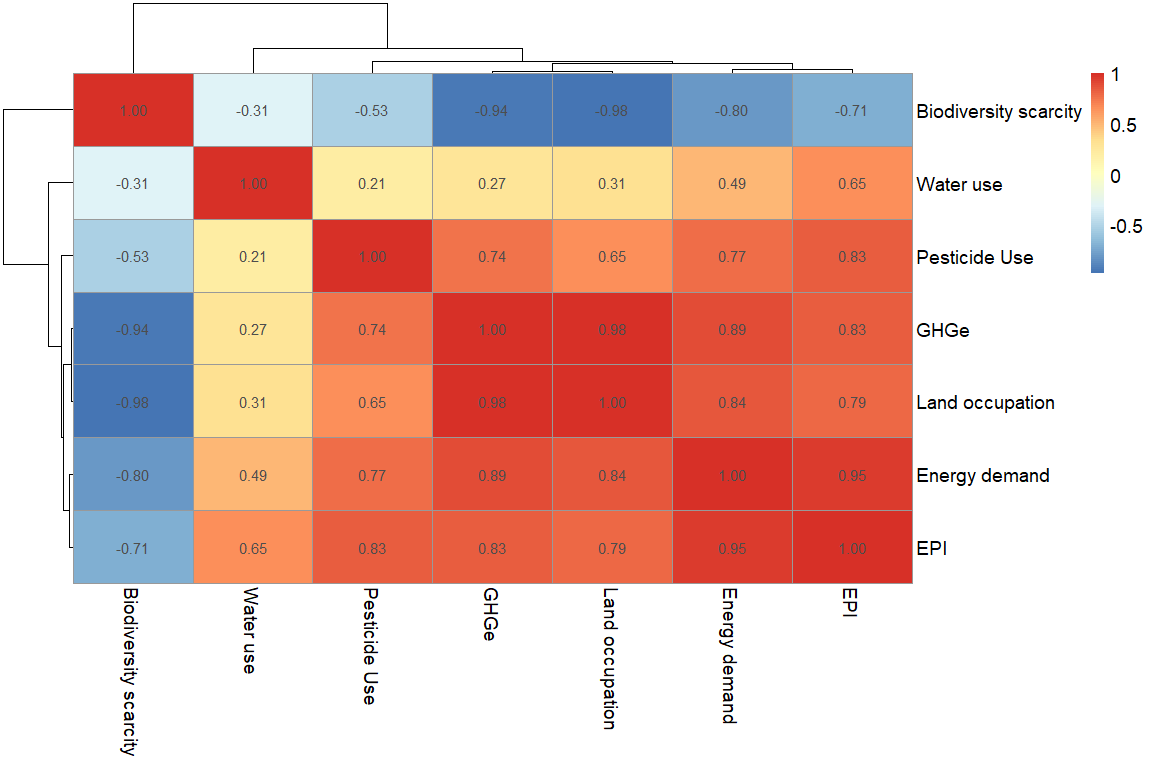


Abbreviations: EI ecological infrastructures; EPI, environmental pressures index; GHG, greenhouse gas emissions. Biodiversity scarcity is (100 - Ecological infrastructures) to facilitate reading and ensure that all indicators point in the same direction.

# Supplemental Table 1: Comparison of participants in the BioNutriNet Project with and without 3 dietary evaluations^1^:

| Variable | without follow-up | with follow-up | P |
| --- | --- | --- | --- |
| %women | 76.9 | 69.99 | <.0001 |
| Gender |  |  |  |
| Age, y | 52.36 (14.69) | 56.21 (11.87) | <.0001 |
| Energy intake, Kcal/d | 1995.16 (637.61) | 2013.72 (608.67) | 0.0195 |
| Smoking status |  |  |  |
| former smokers | 39.83 | 41.88 | <.0001 |
| current smoker | 11.51 | 9.03 |  |
| non-smokers | 48.66 | 49.09 |  |
| Occupation status |  |  |  |
| unemployed | 4.5 | 3.09 | <.0001 |
| retired | 35.13 | 42.11 |  |
| employees | 14.26 | 11.35 |  |
| independent | 1.87 | 1.46 |  |
| intermediate professions | 14.71 | 14.7 |  |
| manager and intellectual professions | 20.98 | 21.09 |  |
| never employed | 7.56 | 4.5 |  |
| Monthly income (%) |  |  |  |
| <1200€ | 12.73 | 8.99 |  |
| 1200-1800 | 23.97 | 21.2 | <.0001 |
| 1800-3700 | 44.89 | 48.74 |  |
| >3700€ | 11.94 | 15.99 |  |
| NA | 6.48 | 5.09 |  |
| Living status (%) |  |  |  |
| Cohabiting | 73.77 | 75.59 | 0.0010 |
| Single | 11.13 | 10.01 |  |
| Education (%) |  |  |  |
| < High school diploma | 21.51 | 20.86 | 0.41 |
| High school | 63.82 | 64.54 |  |
| Post-secondary graduate | 14.67 | 14.6 |  |
| Physical activity level (%) |  |  |  |
| NA | 11.13 | 10.01 | 0.001 |
| Low | 32.99 | 35.05 |  |
| Medium | 36.42 | 36.31 |  |
| High | 32.99 | 35.05 |  |
| Plant-based protein/total protein | 0.34 (0.15) | 0.33 (0.13) | <.0001 |
| Share of organic food in the diet | 0.30 (0.27) | 0.30 (0.27) | 0.80 |
| GHGe (kgCO2eq/d) | 4.02 (2.53) | 4.14 (2.39) | <.0001 |
| Energy demand (kJ/d) | 17.51 (7.61) | 17.90 (7.45) | <.0001 |
| Land occupation (m2/d) | 10.51 (6.90) | 10.81 (6.41) | 0.0005 |
| Water use (m3/d) | 0.58 (0.39) | 0.59 (0.38) | 0.0578 |
| Pesticide use (m²/d) | 24.91 (12.62) | 25.58 (12.32) | <.0001 |
| EI | 0.82 (0.55) | 0.84 (0.52) | <.0001 |

**^1^ Values are mean (SD) or %, p referred to t-test or Chi² test**

# Supplemental Table 2: Number of individuals for each profile identified for each environmental pressure studied across profiles of EPI

|  | Number of participants | Distribution in EPI profiles | |  | |
| --- | --- | --- | --- | --- | --- |
|  |  | Profile 1 | Profile 2 | Profile 3 | Profile 4 |
| GHGe trajectories |  |  |  |  |  |
| Profile 1 | 23 | 13 (56.52) | 0 (0) | 6 (26.09) | 4 (17.39) |
| Profile 2 | 8317 | 66 (0.79) | 41 (0.49) | 8090 (97.27) | 120 (1.44) |
| Profile 3 | 130 | 68 (52.31) | 0 (0) | 62 (47.69) | 0 (0) |
| Profile 4 | 435 | 4 (0.92) | 0 (0) | 263 (60.46) | 168 (38.62) |
| Energy trajectories |  |  |  |  |  |
| Profile 1 | 208 |  | 36 (17.31) | 172 (82.69) | 0 (0) |
| Profile 2 | 8304 | 70 (0.84) | 5 (0.06) | 8095 (97.48) | 134 (1.61) |
| Profile 3 | 149 | 79 (53.02) | 0 (0) | 67 (44.97) | 3 (2.01) |
| Profile 4 | 244 | 2 (0.82) | 0 (0) | 87 (35.66) | 155 (63.52) |
| EI trajectories |  |  |  |  |  |
| Profile 1 | 56 | 2 (3.57) | 10 (17.86) | 42 (75) | 2 (3.57) |
| Profile 2 | 242 | 69 (28.51) | 0 (0) | 171 (70.66) | 2 (0.83) |
| Profile 3 | 7943 | 74 (0.93) | 31 (0.39) | 7685 (96.75) | 153 (1.93) |
| Profile 4 | 664 | 6 (0.9) | 0 (0) | 523 (78.77) | 135 (20.33) |
| Pesticides use trajectories |  |  |  |  |  |
| Profile 1 | 973 | 84 (8.63) | 0 (0) | 850 (87.36) | 39 (4.01) |
| Profile 2 | 6972 | 61 (0.87) | 38 (0.55) | 6839 (98.09) | 34 (0.49) |
| Profile 3 | 960 | 6 (0.63) | 3 (0.31) | 732 (76.25) | 219 (22.81) |
| Land occupation trajectories |  |  |  |  |  |
| Profile 1 | 7416 | 137 (1.85) | 10 (0.13) | 7166 (96.63) | 103 (1.39) |
| Profile 2 | 703 | 0 (0) | 31 (4.41) | 672 (95.59) | 0 (0) |
| Profile 3 | 786 | 14 (1.78) | 0 (0) | 583 (74.17) | 189 (24.05) |
| Water use trajectories |  |  |  |  |  |
| Profile 1 | 2839 | 30 (1.06) | 17 (0.6) | 2689 (94.72) | 103 (3.63) |
| Profile 2 | 6066 | 121 (1.99) | 24 (0.4) | 5732 (94.49) | 189 (3.12) |

Number of participants (%)
EPI: environmental pressures index; EI: Ecological infrastructure

# Supplemental Figure 1: Participants’ selection flowchart based on the three food frequency questionnaires from the NutriNet-Santé cohort.


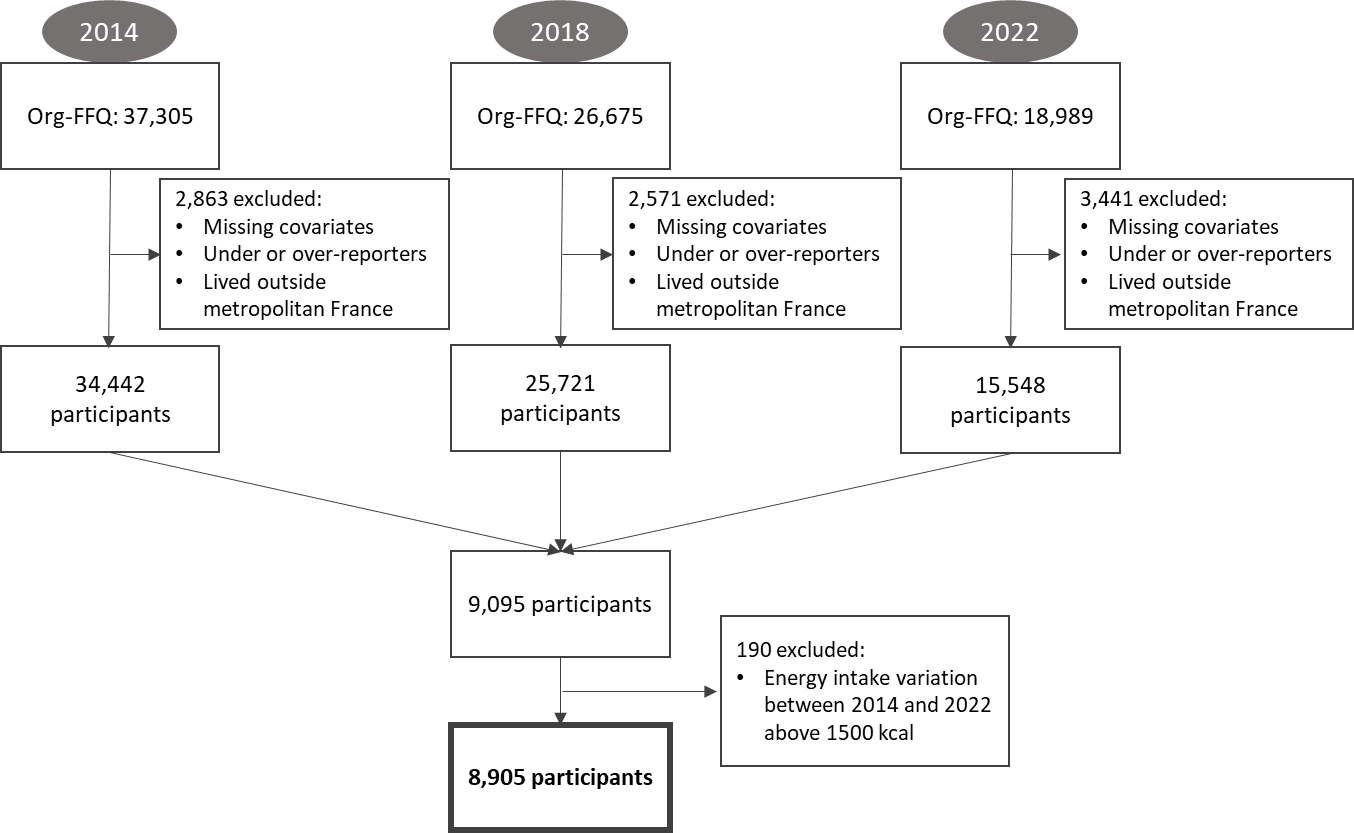


# Supplemental Figure 2: Correlation between environmental indicators (pressures/d)


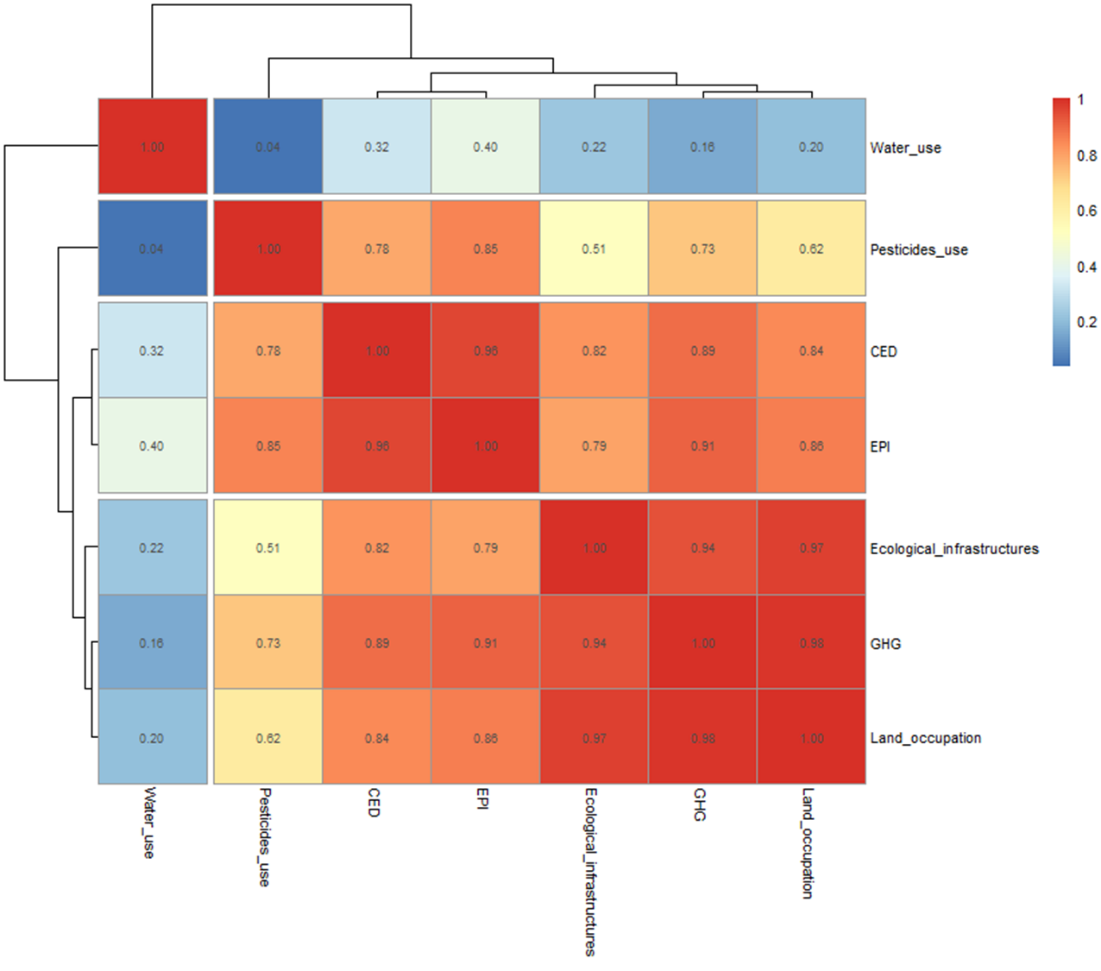


Abbreviations: EPI, Environmental Pressures Index; GHG, greenhouse gas

# Supplemental Figure 3: Specific trajectories for each individual environmental indicator (NutriNet-Santé study FFQ, 2014-2022, N = 8,905)^1^

^
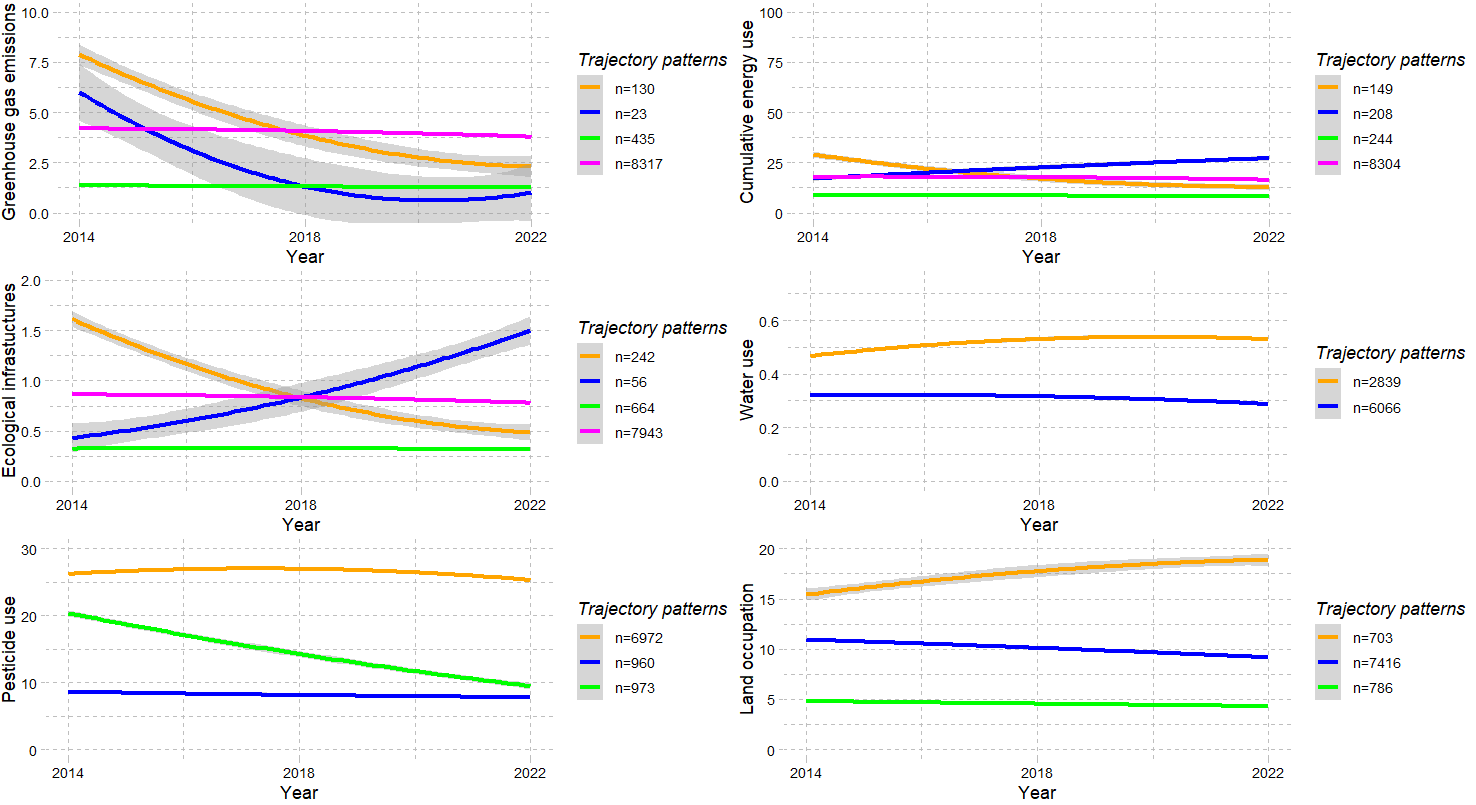
1^Trajectories identified for each indicator are adjusted for age, gender, and energy intake

References

1. Council Regulation (EC) No 2018/848 of 30 may 2018 on on organic production and labelling of organic products and repealing Council Regulation (EC) No 834/2007. (n.d.). Retrieved 24 April 2021, from https://eur-lex.europa.eu/legal-content/EN/TXT/?uri=CELEX%3A02018R0848-20201114

2. FAOSTAT. (2024). Detailed trade matrix. https://www.fao.org/faostat/en/#data/TM

3. Couturier, C., Charru, M., Doublet, S., & Pointereau, P. (2016). Afterres2050_version2016. https://afterres2050.solagro.org/wp-content/uploads/2015/11/solagro_afterres2050_version2016.pdf

4. FAOSTAT. (2024). Food Balances. https://www.fao.org/faostat/en/#data/FBS

5. FAOSTAT. (2024). Data. https://www.fao.org/faostat/en/#data

6. Pointereau, P., Langevin, B., & Gimaret, M. (2012, July). DIALECTE, a comprehensive and quick tool to assess the agro-environmental performance of farms. 10th European IFSA Symposium: Producing and reproducing farming systems. http://ifsa.boku.ac.at/cms/index.php?id=ifsa2012

7. Colomb, V., Amar, S. A., Mens, C. B., Gac, A., Gaillard, G., Koch, P., Mousset, J., Salou, T., Tailleur, A., & Werf, H. M. G. van der. (2015). AGRIBALYSE®, the French LCI Database for agricultural products: High quality data for producers and environmental labelling. OCL, 22(1), Article 1. https://doi.org/10.1051/ocl/20140047

8. Gac, A., Tailleur, A., & Dauguet, S. (2020). Allocation des impacts environnementaux à un produit ou à une activité agricole. https://www.arvalis.fr/sites/default/files/imported_files/___2-152978628971939315.pdf

9. PAN Europe & Pesticides Action Network Europe. (2005). Danish Pesticide Use Reduction Programme—To Benefit the Environment and the Health.

10. Ministère de l’Agriculture et de l’Alimentation. (2018). Indicateur de fréquence de traitements phytopharmaceutiques (IFT), Guide méthodologique Version 3 Avril 2018. https://agriculture.gouv.fr/indicateur-de-frequence-de-traitements-phytosanitaires-ift

11. Gomiero, T., Pimentel, D., & Paoletti, M. G. (2011). Environmental impact of different agricultural management practices: Conventional vs. Organic agriculture. Crit Rev Plant Sci., 30, 95–124.

12. Geiger, F., Bengtsson, J., Berendse, F., Weisser, W. W., Emmerson, M., Morales, M. B., Ceryngier, P., Liira, J., Tscharntke, T., Winqvist, C., Eggers, S., Bommarco, R., Pärt, T., Bretagnolle, V., Plantegenest, M., Clement, L. W., Dennis, C., Palmer, C., Oñate, J. J., … Inchausti, P. (2010). Persistent negative effects of pesticides on biodiversity and biological control potential on European farmland. Basic and Applied Ecology, 11(2), 97–105.

13. Pfister, S., Koehler, A., & Hellweg, S. (2009). Assessing the Environmental Impacts of Freshwater Consumption in LCA. Environmental Science & Technology, 43(11), 4098–4104. https://doi.org/10.1021/es802423e

14. Boulay, A.-M., Bare, J., Benini, L., Berger, M., Lathuillière, M. J., Manzardo, A., Margni, M., Motoshita, M., Núñez, M., Pastor, A. V., Ridoutt, B., Oki, T., Worbe, S., & Pfister, S. (2018). The WULCA consensus characterization model for water scarcity footprints: Assessing impacts of water consumption based on available water remaining (AWARE). The International Journal of Life Cycle Assessment, 23(2), 368–378. https://doi.org/10.1007/s11367-017-1333-8

15. Hoekstra, A. Y., Chapagain, A., Aldaya, M. M., & Mekonnen, M. M. (2009). Water Footprint Manual: State of the Art 2009, Water Footprint Network. www.waterfootprint.org/downloads/WaterFootprintManual2009.pdf

16. Pfister, S., Koehler, A., & Hellweg, S. (2009). Assessing the Environmental Impacts of Freshwater Consumption in LCA. Environmental Science & Technology, 43(11), 4098–4104. https://doi.org/10.1021/es802423e

17. Hoekstra, A. Y. (Ed.). (2011). The water footprint assessment manual: Setting the global standard. Earthscan.

18. WULCA. (2021). AWARE (Available WAter REmaining) Mission and Goals. WULCA. https://wulca-waterlca.org/aware/

19. Banque nationale des prélèvements quantitatifs en eau. (n.d.). Eaufrance | Le service public d’information sur l’eau. Eaufrance. Retrieved 3 March 202 C.E., from https://www.eaufrance.fr/

20. Agreste, la statistique agricole. (n.d.). Recensement agricole 2020—Surface moyenne des exploitations agricoles en 2020: 69 hectares en France métropolitaine et 5 hectares dans les DOM|Agreste, la statistique agricole. Retrieved 3 March 2025, from https://agreste.agriculture.gouv.fr/agreste-web/disaron/Pri2213/detail/

21. Nair, S., Johnson, J., & Wang, C. (2013). Efficiency of Irrigation Water Use: A Review from the Perspectives of Multiple Disciplines. Agronomy Journal, 105(2), 351–363. https://doi.org/10.2134/agronj2012.0421

22. Agreste, la statistique agricole. (2020). Enquête pratiques culturales en grandes cultures et prairies 2017—Principaux résultats (Version modifiée)| (Nos. 2020–9; Chiffres et Données N°). https://agreste.agriculture.gouv.fr/agreste-web/disaron/Chd2009/detail/

23. Huijbregts, M. (2016). ReCiPe 2016—A harmonized life cycle impact assessment method at midpoint and endpoint level Report I: Characterization [RIVM Report].

24. Telepac. (n.d.). Formulaires et notices 2022- Déclaration des surfaces d’intérêt écologique (SIE) » (Métropole). Retrieved 3 March 2024, from https://www.telepac.agriculture.gouv.fr/telepac/html/public/aide/formulaires-2022.html

25. Solagro. (n.d.). Carte ‘pesticides’—Adonis—Solagro. Retrieved 3 March 2025, from https://solagro.org/nos-domaines-d-intervention/agroecologie/carte-pesticides-adonis

26. Prévost, M.-C., Ménard, J.-L., & Leclerc, M.-C. (2012). La maîtrise de la consommation d’eau en élevage bovin laitier. https://www.rmtelevagesenvironnement.org/backoffice/uploads/46_outil_maitrise_conso_eau_elevage_bovin_laitier.pdf

27. Consommations d’eau en élevage: Entre sobriété et résilience. (2022, August 12). Institut de l’Élevage. https://idele.fr/detail-article/consommations-deau-en-elevage-entre-sobriete-et-resilience
